# Factors associated with success in transition care services among older people in Australia

**DOI:** 10.1186/s12877-020-01914-z

**Published:** 2020-11-23

**Authors:** Monica Cations, Catherine Lang, Maria Crotty, Steven Wesselingh, Craig Whitehead, Maria C. Inacio

**Affiliations:** 1 South Australian Health and Medical Research Institute, PO Box 11060, Adelaide, SA 5001 Australia; 2 College of Medicine and Public Health, Flinders University, Adelaide, SA Australia

**Keywords:** Transition care, Intermediate care, Hospital avoidance, Rehabilitation, Older adults

## Abstract

**Background:**

The Australian Transition Care Program (TCP) is a national intermediate care service aiming to optimise functional independence and delay entry to permanent care for older people leaving hospital. The aim of this study was to describe the outcomes of TCP and identify demographic and clinical factors associated with TCP ‘success’, to assist with clinical judgements about suitable candidates for the program.

**Method:**

We conducted a descriptive cohort study of all older Australians accessing TCP for the first time between 2007 and 2015. Logistic regression models assessed demographic and clinical factors associated with change in performance on a modified Barthel Index from TCP entry to discharge and on discharge to community. Fine-Gray regression models estimated factors associated with transition to permanent care within 6 months of TCP discharge, with death as a competing event.

**Results:**

Functional independence improved from entry to discharge for 46,712 (38.4%) of 124,301 TCP users. Improvement was more common with younger age, less frailty, shorter hospital stay prior to TCP, and among women, those without a carer, living outside a major city, and without dementia. People who received TCP in a residential setting were far less likely to record improved functional impairment and more likely to be discharged to permanent care than those in a community setting. Discharge to community was more common with younger age and among women and those without dementia. Nearly 12% of community TCP and 63% of residential TCP users had transitioned to permanent care 6 months after discharge. Entry to permanent care was more common with older age, higher levels of frailty, and among those with dementia.

**Conclusions:**

More than half of TCP users are discharged to home and remain at home after 6 months. However, residential-based TCP may have limited efficacy. Age, frailty, carer status, and dementia are key factors to consider when assessing program suitability. Future studies comparing users to a suitably matched control group will be very helpful for confirming whether the TCP program is meeting its aims.

**Supplementary Information:**

The online version contains supplementary material available at 10.1186/s12877-020-01914-z.

## Background

Supporting older people to transition between health and aged care services is a global challenge [1]. The high costs of both hospital and permanent residential aged care (PRAC) [2] has generated increased investment in programs that support older people to live at home for as long as possible. However, hospitalisations can trigger premature entry to PRAC if sufficient functional recovery to allow return to home has not been achieved [3]. A national Transition Care Program (TCP) was introduced in Australia in 2005 to improve integration of health and aged care services for older people, reduce hospital length of stay, optimise functional independence, and delay entry to PRAC [4].

The Australian TCP is a large-scale intermediate care service that intends to provide continuing quality care in a less intensive (and costly) setting than hospitals. Older people leaving hospital are eligible for up to 18 weeks of TCP [5] delivered in residential settings (i.e. in nursing homes) or in the person’s home. The decision about where the person receives TCP is at the discretion of the clinician though also depends on the availability of places [3]. A plan for services is developed by the TCP provider in collaboration with the person, and is regularly reviewed. Services delivered during TCP are flexible and customised to the individual, but typically include low-intensity therapies to improve physical, cognitive, and psychosocial functioning [4]. This might include physiotherapy, occupational therapy, social work support, nursing care, and/or case management, funded on an “occupied place day” basis [3]. The services are accessed either in the residential facility (if the person is placed in residential TCP), at home, or in hospital or community clinics. TCP is fully integrated into the Australian aged care system and is delivered by state-based public health services. At 1 January 2019 there were 4000 operational TCP places / packages across all regions with an average occupancy rate of 85% [6, 7].

Despite being a pillar of the aged care system in Australia, very few studies have evaluated TCP. Internationally, studies of the utility of transition care and other types of intermediate or post-acute care report inconsistent results partly because of differences in service features and populations [8–13]. Moreover very little research has assessed factors that impact the success of TCP. A lack of research means that clinicians rely on professional opinion to select candidates for the program, which often differ. This reduces the efficiency and effectiveness of care [14]. A descriptive evaluation of the Australian TCP from October 2006 to March 2007 reported that older age, residential-based TCP, greater dependence at TCP admission, and fewer allied health hours used during TCP were associated with admission to residential aged care within 6 months [5]. However, these conclusions were based on a small number of TCP users (*n* = 2443) during the TCP roll out, when limited infrastructure was in place. No comprehensive investigation of factors associated with TCP outcomes has been carried out. Given that the Australian TCP is a national program with Government expenditure exceeding $110 million in the 2016/17 financial year [2], detailed examination of the program and who it benefits is warranted.

The aim of this study is therefore to examine TCP users to (a) describe the common outcomes of receiving TCP, and (b) identify factors associated with TCP ‘success’, defined here as improved functional independence from admission to discharge, discharge to home, and continued residence at home at 6-months post-discharge.

## Methods

### Design and participants

We conducted a descriptive cohort study using data from the Registry of Senior Australians (ROSA) cohort of all older Australians who accessed government-subsidised aged care services from 1997 [15, 16]. We used de-identified linked data from the Australian Institute of Health and Welfare National Aged Care Data Clearinghouse, including dates of entry to and discharge from the TCP program, discharge location after TCP, functional independence at entry and discharge, and date of entry to PRAC. The National Death Index dataset provided dates and causes of death. The Pharmaceutical Benefits Scheme dataset provided information on medicines.

Older people are eligible for TCP if they are: (a) assessed by an Aged Care Assessment Program assessor; (b) an inpatient in a public or private Australian hospital, or receiving sub-acute care; (c) medically stable and ready for discharge from acute or sub-acute care; (d) otherwise eligible for residential aged care, and; (e) have the capacity to benefit from low-intensity therapies over a maximum of 18 weeks, according to clinical judgement. Participants were included in this study if they were (a) non-Indigenous; (b) aged 65 years or older at TCP entry; and (c) used TCP for the first time between January 12,007 to December 312,015. This date range begins when the national program roll out had been completed and ends at the end of the last full year available in our data capture. Aboriginal and Torres Strait Islander older people were not available in our data capture.

### Measures

#### Outcomes of interest

Functional independence (Outcome 1) is measured at TCP admission and discharge using the modified Barthel Index (mBI). The mBI is a is a 10-item measure in which clinical staff rate the person’s ability to independently perform self-care tasks (Table S1). Scores range from zero (complete dependence) to 100 (complete independence) [17] and are categorised for clinical use. For this study we considered that an individual had improved from TCP entry to discharge where they moved ‘up’ one or more categories (e.g. from ‘severe dependence’ to ‘mild dependence’). Conversely, those who moved ‘down’ one or more categories were considered to have deteriorated. Such moves were considered to indicate clinically relevant changes in function over time. A discharge mBI was not conducted for those who were prematurely discharged from TCP due to hospital readmission, so these users were categorised as having deteriorated.

Discharge location (Outcome 2) was recorded at discharge and was categorised as to home or other (PRAC, hospital, another TCP episode, other location). People who died during their TCP episode were excluded from analysis of both Outcome 1 and 2. Dates of admission to permanent residential care were used to categorise place of residence at 6 months post-TCP discharge (home or PRAC; Outcome 3), with death considered a competing risk.

#### Variables of interest

Potential factors associated with TCP outcomes included variables found to affect the success of TCP in previous reports [7] plus additional variables considered by the authors as important for rehabilitation. Variables included age, sex, and country of birth (Australia vs other), availability of an unpaid carer (yes/no), region where the individual resided at time of TCP entry [18] (categorised as major city vs regional/remote/rural), length of stay in TCP (in weeks), and length of hospital stay prior to TCP entry (in weeks). Number of comorbid health conditions were determined using the RxRisk-V, [19] a medication-based comorbidity index based on 6 months history of medication dispensing prior to the hospitalisation that preceded TCP entry. Dementia status was determined from both the aged care eligibility assessment and dispensing of medications prescribed for the treatment of Alzheimer’s disease in the 6 months prior to hospitalisation. A previously validated frailty index measure created using aged care eligibility assessment data was also used [20].

### Analysis

All results were stratified by TCP setting (residential, community, or both), given the known clinical and demographic differences between these groups [7]. Multinomial logistic regression modelling was used to identify factors associated with change in mBI score from TCP entry to discharge (Outcome 1) with individuals whose mBI category did not change as the reference category. Binomial logistic regression modelling was used to identify factors associated with discharge to home (Outcome 2). Fine-Gray subdistribution hazard regression modelling was used to identify factors associated with entry to PRAC within six-months of TCP discharge, accounting for the competing risk of death (Outcome 3). Time to PRAC was the difference (in days) between TCP discharge and PRAC entry date; individuals who had not entered PRAC by six-months after TCP discharge were censored.

For all three outcomes, univariate analysis was conducted using α < 0.05 as the cut-off for inclusion in multivariate modelling. Akaike information criteria were inspected to ensure model fit. All models were adjusted for state of residence given the noted geographical variation in the use of TCP in Australia [4]. Missing data (present in < 3.4% of cases) were managed via casewise deletion. All tests were two-sided and adjusted for multiple comparisons using the stepdown Bonferroni method, and α < 0.0125 was considered statistically significant in final models. Collinearity was assessed via inspection of variance inflation factors (VIF); no problematic VIF (> 10) was identified in any model. Proportional hazards assumptions (where applicable) were checked and satisfied via visual inspection of Schoenfeld residual plots. SAS version 9.4 was used for analysis.

## Results

There were 121,954 individuals with first time TCP episodes included in the study (Fig. S1). Of these, 67,339 (54.2%) people received TCP in the community, 42,165 (33.9%) in residential settings, and 14,797 (11.9%) in both places (Table 1). Individuals were on average 82 years old (SD = 7.3 years), 63.4% were women, 33.4% was born outside Australia, and 77.5% were recorded as having a carer at TCP entry.

**Table 1:** Demographic and clinical features of individuals at their first TCP episode (2007 to 2015)

	Missing	*n* (%) or x (SD)			
		All (*n =* 124,301)	TCP setting		
			Community (*n* = 67,339)	Residential (*n* = 42,165)	Both (*n* = 14,797)
**Age (years)**	0	81.9 (7.3)	81.0 (7.1)	83.2 (7.3)	82.2 (7.2)
**Female**	0	78,834 (63.4)	42,748 (63.5)	25,999 (61.7)	10,087 (68.2)
**Born outside Australia**	482	41,303 (33.4)	20,781 (31.0)	16,070 (38.2)	4452 (30.2)
**Has carer**	921	95,946 (77.5)	50,542 (75.4)	34,156 (81.2)	11,248 (76.2)
**Region**	284				
Major city		80,513 (56.9)	39,149 (58.2)	32,840 (78.2)	8524 (57.7)
Regional / Remote		43,504 (35.1)	28,101 (41.8)	9148 (21.8)	6255 (42.3)
**State**	0				
New South Wales		37,843 (30.4)	32,224 (47.9)	3949 (9.4)	1670 (11.3)
Victoria		35,487 (28.6)	8579 (12.7)	21,536 (51.1)	5372 (36.3)
Queensland		22,918 (18.4)	17,216 (25.6)	2621 (6.2)	3081 (20.8)
South Australia		11,465 (9.2)	4929 (7.3)	3680 (8.7)	2856 (19.3)
Western Australia		11,297 (9.1)	1831 (2.7)	8620 (20.4)	846 (5.7)
Tasmania		3144 (2.5)	1438 (2.1)	1322 (3.1)	384 (2.6)
Australian Capital Territory		1692 (1.4)	784 (1.2)	343 (0.8)	565 (3.8)
Northern Territory		455 (0.4)	338 (0.5)	94 (0.2)	23 (0.2)
**Comorbidities (median, IQR)** ^**a**^	0	5 (3–7)	5 (3–7)	5 (3–7)	5 (3–7)
0		5796 (4.7)	3167 (4.7)	1948 (4.6)	681 (4.6)
1–4		45,087 (36.3)	24,376 (36.2)	15,336 (36.4)	5375 (36.3)
5–9		64,910 (52.2)	35,182 (52.3)	21,971 (51.1)	7757 (52.4)
10+		8508 (6.8)	4614 (6.9)	9210 (6.90)	984 (6.7)
**Dementia**	0	18,121 (15.6)	5912 (8.9)	10,701 (25.4)	1508 (10.2)
**Frailty at entry** ^**b**^ **(median, IQR)**	49	0.3 (0.2–0.3)	0.2 (0.2–0.3)	0.3 (0.2–0.3)	0.3 (0.2–0.3)
0		482 (0.4)	293 (0.4)	130 (0.3)	59 (0.4)
0.1–0.19		21,596 (17.4)	13,277 (19.7)	5811 (13.8)	2508 (17.0)
0.2–0.29		62,188 (50.0)	34,022 (50.5)	20,590 (48.9)	7576 (51.2)
0.3+		39,986 (32.2)	19,725 (29.3)	15,613 (37.1)	4648 (31.4)
**Hospital LOS in days (media, IQR)** ^**c**^	197	27 (17–42)	26 (16–41)	28 (19–43)	26 (17–40)
**TCP LOS in days (median, IQR)** ^**d**^	0	55 (29–84)	60 (34–84)	38 (19–65)	79 (53–85)

*IQR* Interquartile range; *LOS* Length of stay; *SD* Standard deviation; *TCP* Transition Care Program

^a^ Medication-based comorbidity index. Does not include dementia

^b^ Based on aged care assessment data, range 0–1. Higher scores denote greater frailty

^c^ > 365 days set to missing

^d^ > 129 excluded

### Improved functional independence

Among 121,596 individuals discharged from TCP, 38.4% recorded improved mBI score, 37.9% did not change, and 23.6% worsened from entry to discharge (Table 2). Factors associated with improved mBI score across all settings were younger age, female sex, not having a carer, living in a regional area, less frailty, shorter stay in hospital prior to TCP entry, and longer stay in TCP (Table 3). People with dementia were less likely to record improvement in all groups.

**Table 2:** Outcomes of first TCP episode, by setting

	*n* (%)			
	All (*n =* 124,301)	TCP setting		
		Community (*n* = 67,339)	Residential (*n* = 42,165)	Both (*n* = 14,797)
**Functional capacity at entry (Barthel) (median, IQR)** ^**a**^	76 (59–87)	83 (72–90)	61 (40–77)	72 (56–82)
Independent	1579 (1.3)	1204 (1.8)	267 (0.6)	108 (0.7)
Slight dependence	16,459 (13.2)	13,194 (19.6)	2088 (5.0)	1177 (8.0)
Moderate dependence	73,136 (58.8)	44,968 (66.8)	19,220 (45.6)	8948 (60.5)
Severe dependence	26,563 (21.4)	6855 (10.2)	15,572 (36.9)	4136 (28.0)
Total dependence	6564 (5.3)	1118 (1.7)	5018 (11.9)	428 (2.9)
**Functional capacity at exit (Barthel) (median, IQR)** ^**a,b**^	(*n =* 121,596) 90 (75–98)	(*n* = 66,674) 95 (87–100)	(*n* = 40,271) 75 (50–89)	(*n* = 14,651) 91 (82–97)
Independent	17,190 (14.1)	14,046 (21.1)	1401 (3.5)	1743 (11.9)
Slight dependence	28,511 (23.5)	19,437 (29.2)	4600 (11.4)	4474 (30.5)
Moderate dependence	35,579 (29.3)	15,263 (22.9)	15,268 (37.9)	5048 (34.5)
Severe dependence	9882 (8.1)	1680 (2.5)	7389 (18.4)	813 (5.6)
Total dependence	4638 (3.8)	981 (1.5)	3334 (8.3)	322 (2.2)
Discharged to hospital	25,796 (21.2)	15,267 (22.9)	8278 (20.6)	2251 (15.4)
**Barthel category change entry to exit (median, IQR)** ^**b**^	(*n =* 121,596)	(*n* = 66,674)	(*n* = 40,271)	(*n* = 14,651)
Deteriorated ^c^	28,750 (23.6)	16,452 (24.7)	9746 (24.2)	2552 (17.4)
No change	46,134 (37.9)	20,269 (30.4)	21,136 (52.5)	4729 (32.3)
Improved	46,712 (38.4)	29,953 (44.9)	9389 (23.3)	7370 (50.3)
**Discharged to**				
Deceased	2705 (2.2)	665 (1.0)	1894 (4.5)	146 (1.0)
Other	6350 (5.1)	3956 (5.9)	1601 (3.8)	784 (5.3)
Hospital	25,769 (20.8)	15,267 (22.7)	8278 (19.6)	2251 (15.2)
PRAC	24,825 (20.0)	1862 (2.8)	21,352 (50.6)	1611 (10.9)
Community	64,190 (52.2)	45,411 (67.8)	8840 (21.4)	9939 (67.8)
Other TCP	435 (0.4)	169 (0.3)	200 (0.5)	66 (0.5)
**Status at 6 months post-discharge**				
PRAC	37,728 (30.4)	7819 (11.6)	26,606 (63.1)	3303 (22.3)
Deceased	10,920 (8.8)	5522 (8.2)	4399 (10.4)	999 (6.8)
Other	75,653 (60.9)	53,998 (80.2)	11,160 (26.5)	10,495 (70.9)

*IQR* Interquartile range; *PRAC* Permanent residential aged care; *SD* Standard deviation; *TCP* Transition care program

^a^ Higher scores denote greater independence, range 0–100

^b^ Excludes individuals who died during TCP episode

^c^ Includes individuals discharged to hospital

**Table 3:** Results of multinominal regression analysis assessing factors associated with improved and worsening Modified Barthel Index scores from entry to exit of first TCP episode, adjusted for stat

	Community (*n* = 65,626) ^a^ aOR (95%CI)		Residential (*n* = 39,625) ^b^ aOR (95%CI)		Both (*n* = 14,469) ^c^ aOR (95%CI)	
	Improved (*n* = 29,446)	Worsened (*n* = 16,209)	Improved (*n* = 9236)	Worsened (*n* = 9588)	Improved (*n* = 7270)	Worsened (*n* = 2522)
Age (years)	0.98 (0.98–0.99)	0.99 (0.99–0.99)	0.99 (0.98–0.99)	0.98 (0.98–0.99)	0.99 (0.98–0.99)	0.99 (0.98–1.00) ^d^
Female	1.10 (1.06–1.14)	0.83 (0.79–0.87)	1.15 (1.09–1.21)	0.83 (0.79–0.88)	1.16 (1.07–1.26)	0.91 (0.82–1.01) ^d^
Born outside Australia	0.94 (0.91–0.98) ^d^	0.99 (0.94–1.04) ^d^	0.96 (0.91–1.12) ^d^	1.06 (1.01–1.12) ^d^	0.93 (0.85–1.01) ^d^	1.09 (0.97–1.22) ^d^
No carer	1.40 (1.34–1.47)	0.99 (0.94–1.05) ^d^	1.23 (1.15–1.31)	1.08 (1.01–1.16) ^d^	1.38 (1.26–1.52)	1.03 (0.90–1.17) ^d^
Regional/remote/rural	1.17 (1.12–1.22)	0.84 (0.80–0.88)	1.40 (1.31–1.50)	1.56 (1.46–1.67)	1.17 (1.07–1.27)	0.80 (0.72–0.90)
Comorbidities	1.01 (1.00–1.01) ^d^	1.00 (0.99–1.01) ^d^	1.00 (1.00–1.01) ^d^	1.00 (0.99–1.01) ^d^	1.00 (0.99–1.02) ^d^	1.01 (0.99–1.02) ^d^
Dementia	0.58 (0.55–0.63)	0.75 (0.69–0.81)	0.70 (0.66–0.75)	0.69 (0.65–0.73)	0.64 (0.56–0.72)	0.66 (0.56–0.78)
Frailty index score ^e^	0.83 (0.81–0.85)	1.11 (1.07–1.15)	0.94 (0.90–0.97)	1.01 (0.97–1.04) ^d^	0.83 (0.78–0.88)	1.03 (0.96–1.11) ^d^
Hospital LOS (weeks)	0.95 (0.94–0.95)	1.03 (1.02–1.04)	0.96 (0.95–0.95)	1.01 (1.00–1.02) ^d^	0.94 (0.93–0.95)	1.01 (1.00–1.03) ^d^
TCP LOS (weeks)	1.07 (1.06–1.08)	0.74 (0.73–0.74)	1.07 (1.07–1.08)	0.90 (0.89–0.91)	1.06 (1.05–1.07)	0.84 (0.83–0.85)

*aOR* Adjusted odds ratio; *CI* Confidence interval; *LOS* Length of stay; *TCP* Transition Care Program

^a^ Likelihood ratio χ^2^ = 23,615.7, DF = 34, *p* < 0.001. Excluded: 1048 with missing data, 665 deceased

^b^ Likelihood ratio χ^2^ = 6190.0, DF = 34, *p* < 0.001. Excluded: 646 with missing data, 1894 deceased

^c^ Likelihood ratio χ^2^ = 2303.6, DF = 34, *p* < 0.001. Excluded: 182 with missing data, 146 deceased

^d^ *p* > 0.05 after correction for multiple hypothesis testing

^e^ Rounded to 0.1 increments; higher scores denote greater frailty

Shorter length of stay in TCP was associated with deteriorating mBI performance in all groups. People with dementia were significantly less likely to record any change in their mBI at discharge than entry in all groups, compared to those without dementia. People living outside a major city were less likely to deteriorate during TCP in community settings or both community and residential settings, but the reverse was true for residential TCP. Country of birth and carer status were not significantly associated with deteriorating mBI performance in any group.

### Discharge to home

Of the 121,596 individuals discharged from TCP, 64,190 (52.2%) were discharged to home, 25,769 (20.8%) were discharged back to hospital, and 24,825 (20.0%) were discharged to PRAC (Table 2). People who received TCP in a residential setting were more likely to be discharged to PRAC (50.6%) than those in a community setting (2.8%) or those who completed TCP in both settings (10.9%). In all TCP settings, likelihood of discharge to home was associated with younger age, female sex, living outside a major city, shorter hospital stay prior to TCP, longer stay in TCP, and lower mBI score at TCP entry (Table 4). People with dementia were less likely to be discharged to home in all groups except those who received community-based TCP. For those who received TCP in a residential setting, there was no significant impact of carer status or frailty on discharge to home.

**Table 4:** Results of binomial logistic regression modelling assessing factors associated with discharge from first TCP episode to community, adjusted for state

	Community (*n* = 65,626) ^a^	Residential (*n* = 39,625) ^b^	Both (*n* = 14,518) ^c^
	aOR (95%CI)	aOR (95%CI)	aOR (95%CI)
Age (years)	0.99 (0.99–0.99)	0.97 (0.97–0.98)	0.98 (0.98–0.99)
Female	1.23 (1.18–1.28)	1.28 (1.21–1.35)	1.22 (1.12–1.32)
Born outside Australia	1.13 (1.09–1.18)	1.19 (1.12–1.26)	–
No carer	1.14 (1.09–1.20)	0.99 (0.93–1.06) ^d^	1.10 (1.01–1.21) ^d^
Regional/remote/rural	1.19 (1.14–1.24)	1.27 (1.18–1.36)	1.41 (1.30–1.53)
Comorbidities	1.00 (1.00–1.01) ^d^	1.00 (0.99–1.01) ^d^	1.01 (0.99–1.02) ^d^
Dementia	0.98 (0.92–1.05) ^d^	0.53 (0.49–0.57)	0.78 (0.69–0.88)
Frailty ^e^	0.87 (0.85–0.90)	0.96 (0.92–1.00) ^d^	0.88 (0.83–0.93)
Hospital LOS (weeks)	0.96 (0.95–0.96)	0.94 (0.93–0.95)	0.96 (0.95–0.97)
TCP LOS (weeks)	1.34 (1.33–1.35)	1.07 (1.07–1.08)	1.21 (1.19–1.22)
mBI score at entry ^f^	1.15 (1.14–1.17)	1.20 (1.18–1.21)	1.14 (1.12–1.16)

*aOR* Adjusted odds ratio; *CI* Confidence interval; *LOS* Length of stay; *mBI* Modified Barthel Index; *TCP* Transition Care Program

^a^ Likelihood ratio χ^2^ = 17,794.6, DF = 18, *p* < 0.001. Excluded: 1048 with missing data, 665 deceased

^b^ Likelihood ratio χ^2^ = 6235.1, DF = 18, *p* < 0.001. Excluded: 646 with missing data, 1894 deceased

^c^ Likelihood ratio χ^2^ = 1893.2, DF = 17, *p* < 0.001. Excluded: 133 with missing data, 146 deceased

^d^ *p* > 0.05 after correction for multiple hypothesis testing

^e^ Rounded to 0.1 increments; higher scores denote greater frailty

^f^ Scaled to 10-point increments; higher scores denote greater independence

### Entry to permanent residential aged care

The cumulative incidence of entering PRAC by 6 months after TCP discharge was 11.7% for community TCP users (95%CI 11.4–11.9%), 63.1% for residential TCP users (95%CI 62.7–63.6%), and 22.4% for those who used TCP in both settings (95%CI 21.7–23.1%) (Fig. S2). In all TCP settings, factors associated with earlier entry to PRAC included older age, having dementia, higher levels of frailty, and higher entry mBI score (Table 5). Longer stay in TCP was associated with reduced likelihood of PRAC entry among community TCP users and those who used TCP in both community and residential settings, while the reverse was true for residential TCP users.

**Table 5:** Results of Fine-Gray subdistribution hazard regression models assessing factors associated with entry to PRAC within six months of discharge from first TCP episode, accounting for competing risk for death and adjusted for state

	Community (*n* = 66,334) ^a^	Residential (*n* = 41,484) ^b^	Both (*n* = 14,661) ^c^
	asHR (95%CI)	asHR (95%CI)	asHR (95%CI)
Age (years)	1.05 (1.05–1.06)	1.01 (1.01–1.02)	1.04 (1.03–1.05)
Female	0.95 (0.91–1.00)	–	0.92 (0.85–0.98) ^d^
Born outside Australia	0.84 (0.80–0.89)	0.94 (0.93–0.96)	–
No carer	0.90 (0.85–0.96)	0.93 (0.90–0.95)	0.95 (0.87–1.03) ^d^
Regional/remote/rural	0.93 (0.88–0.97)	0.84 (0.81–0.86)	0.76 (0.71–0.83)
Comorbidities	0.99 (0.98–1.00) ^d^	1.00 (1.00–1.01) ^d^	–
Dementia	1.87 (1.76–1.99)	1.37 (1.35–1.40)	1.73 (1.58–1.89)
Frailty ^e^	1.18 (1.14–1.22)	1.61 (1.38–1.87)	4.34 (2.53–7.67)
Hospital LOS (weeks)	–	1.02 (1.01–1.02)	1.02 (1.01–1.03)
TCP LOS (weeks)	0.89 (0.88–0.89)	1.01 (1.00–1.02)	0.93 (0.92–0.94)
mBI score at entry ^f^	0.95 (0.94–0.96)	0.99 (0.99–0.99)	0.92 (0.91–0.94)

*asHR* Adjusted subdistribution hazard ratio; *CI* Confidence interval; *LOS* Length of stay; *TCP* Transition Care Program

^a^ Wald χ^2^ = 4948.9, DF = 17, *p* < 0.001. Excluded: 1005 with missing data

^b^ Wald χ^2^ = 5543.0, DF = 17, *p* < 0.001. Excluded: 681 with missing data

^c^ Wald χ^2^ = 1346.1, DF = 16, *p* < 0.001. Excluded: 136 with missing data

^d^ *p* > 0.05 after correction for multiple hypothesis testing

^e^ Rounded to 0.1 increments; higher scores denote greater frailty

^f^ Scaled to 10-point increments; higher scores denote greater independence

## Discussion

Thirty-eight per cent of Australians who accessed TCP from 2007 to 2015 recorded improved functional independence from entry to discharge from the program, while the remainder recorded no change or worsened. A previous national descriptive analysis reported that 76% of TCP users from 2005 to 2013 recorded at least a one point improvement on the mBI from entry to discharge [7]. Using a more conservative system to classify functional improvements aimed to assess the clinical significance of any changes suggests that these may be more limited than previously reported. Nonetheless, just over half of TCP users were discharged from the program to home and more than 60% remained living in at home 6 months later. These figures are consistent with previous reports [7].

As such, a significant number of TCP users are returning home and staying home after hospitalisation when they would otherwise require transition to PRAC. That one-fifth of users die or return to hospital suggests that those referred to TCP are appropriately at-risk for poor outcomes, and that TCP may prevent these. One previous study has also demonstrated an improvement in self-rated quality of life at TCP discharge that is sustained at 6 months post-discharge [21]. However, our work is not a comparative-effectiveness study of the TCP program. Future research comparing outcomes of TCP users to a suitably matched control group will be essential for confirming whether the TCP program is achieving its aims. One small Australian trial (*n* = 320) prior to the TCP roll out reported no difference in mortality, function, or hospital readmissions at 4 months between older people who stayed in hospital while awaiting transfer to PRAC and those who moved to a residential site but continued to receive transition services [10]. This is the only Australian controlled trial of transition care services to date, despite the implementation of and investment in the national TCP.

The Australian TCP program is unique but bears similarity to programs overseas. It is an example of an intermediate care program designed to provide continuity of care and reduce hospital readmission. Evidence to support the efficacy of international programs similar to the Australian residential-based TCP is weak, with no impact on function in earlier trials [8]. This trend is continued here, as people who received TCP in residential settings were far less likely to record functional improvements from TCP entry to discharge, more likely to be discharged to PRAC, and markedly more likely to be living in PRAC six-months after TCP discharge. More than 75% of those who received residential TCP were either living in PRAC or deceased 6 months after they left the TCP program. This is partly owing to their higher rates of dementia, frailty, and functional impairment at TCP entry relative to other groups. In a previous evaluation of TCP (conducted during its national rollout) risk for entry to PRAC within 6 months of TCP discharge was similar among residential TCP users and those who were approved for but did not use TCP [5]. Authors noted the high rate of death within 6 months among residential TCP users (one in five) and hypothesised that inappropriate selection of people who are not medically stable into residential TCP may contribute to poor outcomes. Young [8] also argues that the care philosophy in nursing home settings (i.e. of “doing for”) is antithetical to the rehabilitative aims of intermediate care programs, an inherent structural problem that limits the efficacy of these services. Our results continue to raise questions about the effectiveness of residential TCP in providing pathways from hospital back to community living. This may be especially true for people with dementia, with one Japanese study reporting that only 24.4% of people with dementia in residential TCP are discharged to home [22]. Community-based intermediate care programs, particularly ‘hospital-at-home’ services, have a stronger evidence-base overall with trials showing a positive impact on function and healthcare utilisation [23].

The other aim of this study was to identify demographic and clinical factors associated with success in the TCP program. Outcomes from international trials of intermediate care programs have varied according to the program features [8], delays to TCP admission, [24] and study design [25, 26]. In our sample, some factors improved success on all outcomes and across all TCP settings, including younger age and living outside a major city. Both higher levels of frailty and having dementia were associated with poorer outcomes across TCP settings. The potential utility of rehabilitation and restorative care programs for people with dementia remains an active matter of debate, [27, 28] and health professionals report scepticism about the capacity for recovery where cognitive impairment interferes with therapy participation and adherence [29]. Several studies have demonstrated that people with dementia can benefit from rehabilitation programs similarly to people without dementia [30–33] but do require greater resource use to achieve the same outcomes [34]. We were unable to gauge the level of resource use by individuals in our cohort and compare these according to dementia status; this would be a useful avenue for future research. Identifying a level of cognitive impairment and frailty at which intervention becomes futile or harmful is a well-documented clinical challenge [35] and research that can guide these determinations will be important.

People who did not have a carer at TCP entry were more likely to record functional improvements at discharge and were also less likely to be living in PRAC at 6 months post-discharge, even after controlling for functional capacity at entry and length of stay in TCP. This is surprising given the noted benefits of carer availability for rehabilitation in older people [36–38]. People without a carer may have had better functioning prior to the event that led to their pre-TCP hospitalisation and therefore more capacity to return to independent living after TCP. However, our previous work identified that people with a family carer are less likely to enter the TCP program after approval [39] suggesting that those included here are a selected sample and not broadly representative of older people without a care.

### Strengths and limitations

This is the first ever national evaluation of the full Australian TCP program that accounts for the heterogeneity of individuals accessing these services. The large sample, eight-year timespan, and breadth of data available for assessment are key strengths of this study. The inclusion of all first-time TCP use nationally means that the results are representative of the population, and linked data minimises the risk of misclassified outcomes. Aged care eligibility assessments, from which much of the data used in this study are taken, are systematised and conducted by suitably qualified clinicians, maximising the internal validity of these data.

Limitations include that we were unable to examine factors that have been shown in other studies to impact success of intermediate care services, because they are not included in the ROSA dataset. Such factors include the services each person received during TCP and the extent of their engagement with the program [5], satisfaction with care [40], whether entry to TCP was delayed [24], the reason for hospitalisation prior to TCP entry [5], and the regional availability of TCP programs and professional expertise [4]. We were also not able to assess the impact of other potentially important factors including access to housing, income, and socioeconomic status. In addition, data for people who were approved for but did not receive TCP services were not available; we have previously reported that this occurs in approximately 22% of cases [39].

Where individuals used TCP in both community and residential settings, it was not possible for us to determine which setting they attended first. There are likely to be important differences between those who attended residential TCP before community TCP and those who followed the opposite path, especially given the marked differences between community and residential TCP users noted here. Finally, the large size of our sample has produced some weak but statistically significant associations even after corrections for multiple testing were applied. The strength of these associations should be considered.

## Conclusion

Results of this population-based study suggest that while a minority of TCP users record improved functional independence from entry to discharge, more than half are discharged to home and over 60% remain living at home after 6 months. However, success is much less common among people who use residential-based TCP. This data can guide resource allocation and encourage clinicians to be discerning when referring for residential TCP. Success in TCP is more common with younger age, shorter hospital stay prior to TCP entry, lower levels of frailty, and without dementia. These findings can be used as a guide for clinicians when assessing the suitability of candidates for TCP. More evidence is required to ensure TCP is meeting its aims, particularly research that compares outcomes of TCP users to a suitably matched control group. Such work would be complemented by qualitative enquiry to examine the extent to which clinicians consider these factors in determining eligibility for TCP, and the influence on placement availability.

## Supplementary Information

**Additional file 1: Fig. S1**. Consort diagram. **Fig. S2**. Cumulative incidence of entering permanent residential aged care within six months of discharge from Transition Care Program. **Table S1**. Modified Barthel Index used at entry to and exit from Transition Care Program. **Table S2**. Results of univariate analyses for all outcomes.

## Data Availability

The datasets used and analysed during the current study are available upon application to the ROSA team.

## Abbreviations

mBI: Modified barthel index
PRAC: Permanent residential aged care
ROSA: Registry of senior Australians
TCP: Transition care program
VIF: Variance inflation factor
