# Supplementary material for: Factors associated with success in transition care services among older people in Australia
Source: BMC Geriatr. 2020 Nov 23;20:496. doi: 10.1186/s12877-020-01914-z (PMC7686713; doi:10.1186/s12877-020-01914-z)
Supplement: Supplementary file 1 — Additional file 1: Fig. S1. Consort diagram. Fig. S2. Cumulative incidence of entering permanent residential aged care within six months of discharge from Transition Care Program. Table S1. Modified Barthel Index used at entry to and exit from Transition Care Program. Table S2. Results of univariate analyses for all outcomes. [file 12877_2020_1914_MOESM1_ESM.docx]

**Supplementary Material**


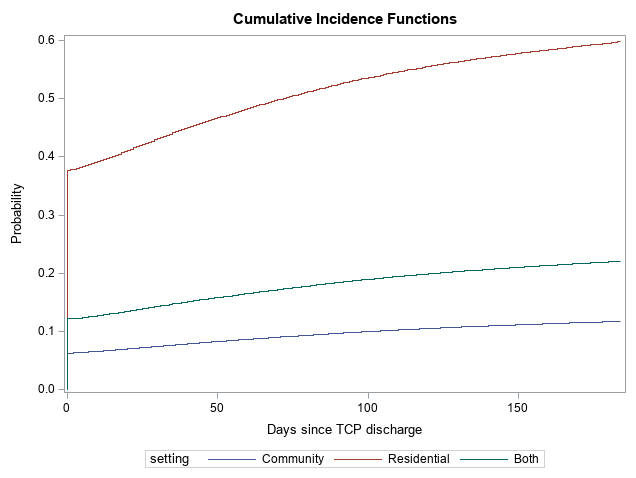


Figure S1. Consort diagram.

**All people accessing TCP from program inception (Oct 2005) - 31 Dec 2015**

*n*=134,846

**No prior ACAT or ACAT>60 days before TCP start**

*n*=4906

**TCP episode ≥130 days**

*n*=359

**TCP in 2005 or 2006**

*n*=3085

**With prior ACAT data**

*n*=129,940

**TCP episode <130 days**

*n*=129,581

**TCP from January 1 2007 to 31 December 2015**

*n*=126,496

**Entered TCP from PRAC**

*n*=2,182

**Entered TCP from community**

*n*=124,314

**TCP setting unknown**

*n*=13

**Included in analysis**

*n*=124,301

Figure S2. Cumulative incidence of entering permanent residential aged care within six months of discharge from Transition Care Program.

Table S1. Modified Barthel Index used at entry to and exit from Transition Care Program.

| *Domain* | *Score* | *Description* |
| --- | --- | --- |
| *Chair/bed transfers* | 0 | Unable to participate in a transfer. Two attendants are required to transfer the care recipient with or without a mechanical device. |
|  | 3 | Able to participate but maximum assistance of one other person is required in all aspects of the transfer. |
|  | 8 | The transfer requires the assistance of one other person. Assistance may be required in any aspect of the transfer. |
|  | 12 | The presence of another person is required either as a confidence measure, or to provide supervision for safety. |
|  | 15 | The care recipient can safely approach the bed walking or in a wheelchair, lock brakes, lift footrests, or position walking aid, move safely to bed, lie down, come to a sitting position on the side of the bed, change the position of the wheelchair, transfer back into it safely and/or grasp aid and stand. The care recipient must be independent in all phases of this activity. |
| *Ambulation* | 0 | Dependent in ambulation. |
|  | 3 | Constant presence of one or more assistant is required during ambulation. |
|  | 8 | Assistance is required with reaching aids and/or their manipulation. One person is required to offer assistance. |
|  | 12 | The care recipient is independent in ambulation but unable to walk 50 metres without help, or supervision is needed for confidence or safety in hazardous situations. |
|  | 15 | The care recipient must be able to wear braces if required, lock and unlock these braces, assume standing position, sit down, and place the necessary aids into position for use. The care recipient must be able to use crutches, canes, or a walkarette, and walk 50 metres without help or supervision. |
| *Stair climbing* | 0 | The care recipient is unable to climb stairs. |
|  | 2 | Assistance is required in all aspects of stair climbing, including assistance with walking aids. |
|  | 5 | The care recipient is able to ascend/descend but is unable to carry walking aids and needs supervision and assistance. |
|  | 8 | Generally no assistance is required. At times supervision is required for safety due to morning stiffness, shortness of breath, etc. |
|  | 10 | The care recipient is able to go up and down a flight of stairs safely without help or supervision. The care recipient is able to use hand rails, cane or crutches when needed and is able to carry these devices as he/she ascends or descends. |
| *Toilet transfers* | 0 | Fully dependent in toileting. |
|  | 2 | Assistance required in all aspects of toileting. |
|  | 5 | Assistance may be required with management of clothing, transferring, or washing hands. |
|  | 8 | Supervision may be required for safety with normal toilet. A commode may be used at night but assistance is required for emptying and cleaning. |
|  | 10 | The care recipient is able to get on/off the toilet, fasten clothing and use toilet paper without help. If necessary, the care recipient may use a bed pan or commode or urinal at night, but must be able to empty it and clean it. |
| *Bowel control* | 0 | The care recipient is bowel incontinent. |
|  | 2 | The care recipient needs help to assume appropriate position, and with bowel movement facilitatory techniques. |
|  | 5 | The care recipient can assume appropriate position, but cannot use facilitatory techniques or clean self without assistance and has frequent accidents. Assistance is required with incontinence aids such as pads, etc. |
|  | 8 | The care recipient may require supervision with the use of suppository or enema and has occasional accidents. |
|  | 10 | The care recipient can control bowels and has no accidents, can use suppository, or take an enema when necessary. |
| *Bladder control* | 0 | The care recipient is dependent in bladder management, is incontinent, or has indwelling catheter. |
|  | 2 | The care recipient is incontinent but is able to assist with the application of an internal or external device. |
|  | 5 | The care recipient is generally dry by day, but not at night and needs some assistance with the devices. |
|  | 8 | The care recipient is generally dry by day and night, but may have an occasional accident or need minimal assistance with internal or external devices. |
|  | 10 | The care recipient is able to control bladder day and night, and/or is independent with internal or external devices. |
| *Bathing* | *0* | Total dependence in bathing self. |
|  | *1* | Assistance is required in all aspects of bathing, but care recipient is able to make some contribution. |
|  | *3* | Assistance is required with either transfer to shower/bath or with washing or drying; including inability to complete a task because of condition or disease, etc. |
|  | *4* | Supervision is required for safety in adjusting the water temperature, or in the transfer. |
|  | *5* | The care recipient may use a bathtub, a shower, or take a complete sponge bath. The care recipient must be able to do all steps of whichever method is employed without another person being present. |
| *Dressing* | 0 | The care recipient is dependent in all aspects of dressing and is unable to participate in the activity. |
|  | 2 | The care recipient is able to participate to some degree, but is dependent in all aspects of dressing. |
|  | 5 | Assistance is needed in putting on, and/or removing any clothing. |
|  | 8 | Only minimal assistance is required with fastening clothing such as buttons, zips, bra, shoes, etc. |
|  | 10 | The care recipient is able to put on, remove, corset, braces, as prescribed. |
| *Personal hygiene* | *0* | The care recipient is unable to attend to personal hygiene and is dependent in all aspects. |
|  | *1* | Assistance is required in all steps of personal hygiene, but care recipient able to  make some contribution. |
|  | *3* | Some assistance is required in one or more steps of personal hygiene |
|  | *4* | Care recipient is able to conduct his/her own personal hygiene but requires minimal assistance before and/or after the task. |
|  | *5* | The care recipient can wash his/her hands and face, comb hair, clean teeth and shave. A male care recipient may use any kind of razor but must insert the blade, or  plug in the razor without help, as well as retrieve it from the drawer or cabinet. A  female care recipient must apply her own make-up, if used, but need not braid or style her hair. |
| *Feeding* | 0 | Dependent in all aspects and needs to be fed, nasogastric needs to be administered. |
|  | 2 | Can manipulate an eating device, usually a spoon, but someone must provide active assistance during the meal. |
|  | 5 | Able to feed self with supervision. Assistance is required with associated tasks such as putting milk / sugar into tea, salt, pepper, spreading butter, turning a plate or other “set up” activities. |
|  | 8 | Independence in feeding with prepared tray, except may need meat cut, milk carton opened or jar lid removed, etc. The presence of another person is not required. |
|  | 10 | The care recipient can feed self from a tray or table when someone puts the food within reach. The care recipient must put on an assistive device if needed, cut food, and if desired use salt and pepper, spread butter, etc. |
| Interpretation | | |
| *0-20* | Total dependence | |
| *21-60* | Severe dependence | |
| *61-90* | Moderate dependence | |
| *91-99* | Slight dependence | |
| *100* | Independence | |

Table S2. Results of univariate analyses for all outcomes.

| **Change in mTI score from TCP entry to exit ^a^** | | | | | | |
| --- | --- | --- | --- | --- | --- | --- |
|  | Community  (*n*=66,674) | | Residential  (*n*=40,217) | | Both  (*n*=14,651) | |
|  | Improved  (*n*=29,953) | Deteriorated  (*n*=16,452) | Improved  (*n*=9389) | Deteriorated  (*n*=9746) | Improved  (*n*=7370) | Deteriorated  (*n*=2552) |
|  | **OR (95%CI)** | **OR (95%CI)** | **OR (95%CI)** | **OR (95%CI)** | **OR (95%CI)** | **OR (95%CI)** |
| Age (years) | 0.98 (0.98-0.98) | 0.99 (0.99-1.00) | 0.99 (0.98-0.99) | 0.98 (0.98-0.98) | 0.99 (0.98-0.99) | 0.99 (0.98-1.00) |
| Female | 1.17 (1.13-1.21) | 0.79 (0.76-0.82) | 1.20 (1.14-1.26) | 0.81 (0.78-0.86) | 1.21 (1.12-1.31) | 0.97 (0.78-0.96) |
| Born outside Australia | 0.82 (0.79-0.85) | 0.95 (0.91-0.99) | 0.77 (0.73-0.81) | 0.92 (0.88-0.97) | 0.86 (0.79-0.93) | 1.05 (0.94-1.16) ^b^ |
| No carer | 1.70 (1.63-1.77) | 1.03 (0.98-1.08) ^b^ | 1.50 (1.41-1.59) | 1.15 (1.08-1.23) | 1.65 (1.51-1.80) | 1.04 (0.92-1.19) ^b^ |
| Regional/remote | 1.29 (1.24-1.34) | 0.98 (0.94-1.02) ^b^ | 2.12 (2.00-2.25) | 1.65 (1.56-1.75) | 1.17 (1.09-1.26) | 0.98 (0.89-1.08) ^b^ |
| Comorbidities | 0.98 (0.97-0.99) | 1.08 (1.07-1.09) | 1.01 (1.00-1.02) ^b^ | 1.07 (1.06-1.08) | 0.98 (0.97-0.99) | 1.08 (1.06-1.97) |
| Dementia | 0.44 (0.42-0.47) | 0.79 (0.74-0.85) | 0.52 (0.49-0.56) | 0.58 (0.54-0.61) | 0.50 (0.45-0.57) | 0.57 (0.58-0.79) |
| Frailty ^d^ | 0.80 (0.78-0.82) | 1.13 (1.10-1.16) | 0.90 (0.87-0.94) | 1.02 (0.99-1.06) ^b^ | 0.80 (0.76-0.85) | 1.04 (0.97-1.11) ^b^ |
| Hospital LOS (weeks) | 0.95 (0.95-0.96) | 1.00 (0.99-1.00) ^b^ | 0.95 (0.94-0.95) | 1.00 (0.99-1.01) ^b^ | 0.94 (0.93-0.95) | 1.00 (0.98-1.01) ^b^ |
| TCP LOS (weeks) | 1.04 (1.04-1.05) | 0.74 (0.74-0.75) | 1.08 (1.07-1.08) | 0.91 (0.99-0.91) | 1.04 (1.03-1.05) | 0.84 (0.83-0.85) |
| **Discharge to community** | | | | | | |
|  | Community  (*n*=66,674) | | Residential  (*n*=40,217) | | Both  (*n*=14,651) | |
|  | **OR (95%CI)** | | **OR (95%CI)** | | **OR (95%CI)** | |
| Age (years) | 0.99 (0.98-0.99) | | 0.97 (0.97-0.98) | | 0.98 (0.98-0.99) | |
| Female | 1.34 (1.29-1.38) | | 1.30 (1.24-1.36) | | 1.27 (1.18-1.37) | |
| Born outside Australia | 1.05 (1.01-1.08) ^c^ | | 0.89 (0.85-0.94) | | 0.99 (0.92-1.07) ^b^ | |
| No carer | 1..33 (1.28-1.39) | | 1.42 (1.34-1.50) | | 1.33 (1.22-1.45) | |
| Regional/remote | 1.09 (1.05-1.12) | | 2.06 (1.95-2.17) | | 1.19 (1.11-1.28) | |
| Comorbidities | 0.94 (0.93-0.94) | | 1.02 (1.01-1.03) | | 0.96 (0.95-0.98) | |
| Dementia | 0.79 (0.75-0.94) | | 0.41 (0.38-0.44) | | 0.64 (0.47-0.71) | |
| Frailty ^d^ | 0.79 (0.77-0.81) | | 0.84 (0.81-0.87) | | 0.83 (0.78-0.88) | |
| Hospital LOS (weeks) | 0.98 (0.98-0.99) | | 0.92 (0.92-0.93) | | 0.97 (0.96-0.98) | |
| TCP LOS (weeks) | 1.32 (1.31-1.32) | | 1.08 (1.08-1.09) | | 1.18 (1.17-1.19) | |
| Functional capacity at entry ^e^ | 1.17 (1.16-1.18) | | 1.24 (1.22-1.25) | | 1.11 (1.09-1.13) | |
| **Time to PRAC entry** | | | | | | |
|  | Community  (*n*=67,339) | | Residential  (*n*=42,165) | | Both  (*n*=14,797) | |
|  | **sHR (95%CI)** | | **sHR (95%CI)** | | **sHR (95%CI)** | |
| Age (years) | 1.06 (1.06-1.07) | | 1.02 (1.02-1.02) | | 1.04 (1.04-1.05) | |
| Female | 0.92 (0.87-0.96) | | 0.99 (0.97-1.01) ^b^ | | 0.91 (0.85-0.98) | |
| Born outside Australia | 0.95 (0.90-0.99) ^c^ | | 1.07 (1.05-1.09) | | 1.04 (0.97-1.12) ^b^ | |
| No carer | 0.68 (0.66-0.72) | | 0.82 (0.79-0.84) | | 0.72 (0.66-0.79) | |
| Regional/remote | 0.88 (0.84-0.92) | | 0.68 (0.66-0.69) | | 0.76 (0.71-0.81) | |
| Comorbidities | 1.01 (1.01-1.02) | | 0.98 (0.97-0.98) | | 1.00 (0.99-1.01) ^b^ | |
| Dementia | 2.46 (2.33-2.61) | | 1.58 (1.55-1.60) | | 2.22 (2.04-2.42) | |
| Frailty ^d^ | 1.34 (1.30-1.38) | | 1.08 (1.06-1.09) | | 1.25 (1.19-1.32) | |
| Hospital LOS (weeks) | 1.01 (1.00-1.01) ^d^ | | 1.02 (1.02-1.02) | | 1.02 (1.01-1.02) | |
| TCP LOS (weeks) | 0.88 (0.88-0.89) | | 1.01 (1.00-1.01) | | 0.93 (0.93-0.94) | |
| Functional capacity at entry ^e^ | 0.88 (0.88-0.89) | | 0.96 (0.96-0.97) | | 0.89 (0.88-0.91) | |

CI=Confidence interval; LOS=Length of stay; OR=Odds ratio; PRAC=Permanent residential aged care; sHR=Subdistribution hazard ratio

^a^ Multinomial logistic regression modelling

^b^ *p* > 0.05

^c^ *p* between .0125 and .05.

^d^ Rounded to 0.1 increments

^e^ Scaled to 10-point increments
